# Complex Permittivity of Ex-Vivo Human, Bovine and Porcine Brain Tissues in the Microwave Frequency Range

**DOI:** 10.3390/diagnostics12112580

**Published:** 2022-10-25

**Authors:** Anđela Matković, Anton Kordić, Antonia Jakovčević, Antonio Šarolić

**Affiliations:** 1 FESB, University of Split, HR-21000 Split, Croatia; 2 Department of Neurosurgery, University Hospital Centre Zagreb, HR-10000 Zagreb, Croatia; 3 Department of Pathology and Cytology, University Hospital Centre Zagreb, HR-10000 Zagreb, Croatia

**Keywords:** dielectric properties measurement, complex permittivity, microwave gigahertz (GHz) frequency range, open-ended coaxial probe, biological tissues, white matter, grey matter, cerebellum, human, bovine and porcine brain

## Abstract

Accurate knowledge about the dielectric properties of biological tissues in the microwave frequency range may lead to advancement of biomedical applications based on microwave technology. However, the published data are very scarce, especially for human brain tissues. The aim of this work was to measure and report the complex permittivity of brain white matter, grey matter and cerebellum. Complex permittivity was measured on human, bovine and porcine brain tissues in the microwave frequency range from 0.5 to 18 GHz using an open-ended coaxial probe. The results present a valuable addition to the available data on the brain tissue complex permittivity. Some noticeable variations between the results lead to several conclusions. Complex permittivity variation within the same tissue type of the individual species was comparable to interspecies variation. The difference was prominent between human brains obtained from autopsies, while bovine brains obtained from healthy animals showed very similar complex permittivity. We hypothesize that the difference might have been caused by the basic pathologies of the patients, where the associated therapies could have affected the brain water content. We also examined the effect of excised tissue degradation on its complex permittivity over the course of three days, and the results suggest the gradual dehydration of the samples.

## 1. Introduction

Interaction of electromagnetic field with a biological tissue crucially depends on the tissue’s dielectric properties, i.e., its complex permittivity. The permittivity is a complex quantity that describes the dielectric polarization (i.e., the ability of a material to store the electric energy) and losses in a material exposed to electric or electromagnetic field. Accurate knowledge of the complex permittivity of biological tissues may lead to advancement of medical diagnostic and therapeutic applications, either inventing new ones, or improving the existing ones. Brain tissues are especially interesting in this respect, as they are relatively underrepresented in the published studies reporting biological tissue complex permittivity measurement, yet various beneficial applications are possible when knowing the exact dielectric properties. 

For example, well defined dielectric properties in the microwave frequency range are crucial for microwave imaging and all related procedures relying on microwave electromagnetic (EM) radiation. Accordingly, medical applications would significantly benefit from precise dielectric characterization of biological tissues in the microwave, or more precisely, in the gigahertz (GHz) frequency range. Microwave imaging is based on the perturbation of the EM field inside the human body that occurs as a result of interaction with biological tissues. The applicability of the described imaging procedure on brain tissues is based on the existence of a significant contrast in dielectric permittivity of healthy tissues and pathological tissues or conditions (such as hemorrhage after a stroke) [1]. A great example is a recent paper published by Saied et al. [2] that describes a novel approach of detecting Alzheimer’s disease by comparing the dielectric properties of a healthy brain with a brain sample from a patient with severe Alzheimer’s disease. Another vivid example of this is the newly developed microwave scanner that enables detection and differentiation of a brain stroke (intracranial hemorrhage or ischemia) by means of microwave technology [3,4,5]. A diagnostic approach combining the aforementioned microwave imaging with brain biomarkers could potentially achieve even higher sensitivity and specificity [6]. Another notable example is the possibility of differentiation between benign and malignant tissues, as already described in papers relating to microwave breast imaging [7] and intraoperative margin-assessment devices [8], thus enabling total or near total tumor removal whilst sparing the healthy tissue. Also, an application very much worth mentioning is microwave beamforming for hyperthermia treatment [9] i.e., heating tumors using microwave EM radiation to the fever range temperatures of ca. 40–44 °C [10]. It is a procedure that should be carefully planned, where accurate knowledge of dielectric properties of brain tissues (especially the cerebellum in pediatric patients as the majority of tumors occur in the posterior fossa region that contains the cerebellum) is paramount because this approach utilizes constructive interference of electromagnetic waves and its selective absorption to achieve localized heating only in the target tumor tissue [9]. However, to do successful hyperthermia treatment planning, even non-target tissues should be well defined e.g., cerebrospinal fluid for brain tumor hyperthermia as shown in [10]. Likewise, exact permittivity of brain tissues is also necessary for hyperthermia treatment planning for non-brain head and neck tumors to avoid unnecessary heating of brain tissues [11]. This results in improved patient treatment quality, reproducibility, operator handling and patient safety [12] as well as it being a cost-effective option [13]. One of the more recent hyperthermia studies of the head and neck region showed the importance of the relationship between the absorbed electromagnetic power and tumor response [14], a subject that could be resolved by deeper understanding of physical properties of target tissues including the dielectric properties. 

Microwave ablation, as another microwave-based technique, has been used since 1994 in liver and pancreatic tumors, but was recently used to treat brain and skull-base tumors [15]. Treatment of brain and skull-base tumors with microwave ablation reported in [15] deemed it as a safe and viable alternative for high-risk brain tumor resection surgery. The treatment planning and designing of microwave applicators for both hyperthermia and ablation using computer simulations would be impossible without accurately modeling the dielectric parameters of all tissues involved in the procedure, i.e., both the target and the surrounding tissues. The former was very recently shown in [16] where the microwave hyperthermia treatment of brain tumors was modeled and simulated using finite element method (FEM), with the brain tissue complex permittivity as the input parameter.

Lastly, dielectric properties of human tissues are the crucial data when analyzing the potentially harmful effects of electromagnetic radiation on the human body. In the microwave frequency range, the main harmful effect is the unwanted heating due to deposition of electromagnetic energy into the tissues [17]. In this case, contrary to medical hyperthermia and ablation, the heating is unwanted and thus considered harmful. Nevertheless, the physical mechanism is the same and depends on the tissue complex permittivity.

Research, innovation and implementation of microwave technology applications in medicine greatly benefit from anatomically realistic computer models of the human body [18], which can then be used in computer electromagnetic simulations (such as [19]). The models are built using high-resolution volumetric elements, where each element is assigned its dielectric properties, so that the microwave propagation inside the body can be faithfully simulated using computer simulations. Without the knowledge about dielectric properties measured at various temperatures, at multiple frequencies and on various subjects, it is difficult to know whether the models appropriately reflect the dielectric properties of the tissues. 

The most commonly cited source of dielectric properties for various tissues (including the brain tissues) is still the one from Gabriel [20], from 1996. However, it is actually a compilation of measurement results from different species, mostly based on ovine tissue measurements. The most comprehensive compilation of physical properties of various tissues, which includes complex permittivity, is provided by [21], here referred to as the IT’IS database. The IT’IS database was chosen as our main reference for comparison given that the database is regularly updated with new data as it becomes available (e.g., last update was done in February 2022) and is thus cited by various recent publications on dielectric properties of tissues. However, most of the dielectric properties of tissues in the IT’IS database are still referenced to Gabriel [20], including also the brain tissues measured in this study. Accordingly, the reference dielectric properties of brain tissues have not been updated since 1996 as a result of lack of published data. 

As the measured dielectric data for brain tissues is poorly covered in the literature, especially for human brain tissues, the aim of this work is to report the measurement data for dielectric properties of brain tissues for several species including humans, providing enough supporting information about our measurement protocol and tissue properties. We report here the measurements of complex permittivity of white matter and grey matter (both from cerebrum) and cerebellum of human, bovine and porcine brains at 25 °C, over the frequency range of 0.5 GHz to 18 GHz. We trust that this data will be of use to researchers that aim to realistically model the brain tissues as well as to simply compare the measurement data.

## 2. Materials and Methods

### 2.1. Complex Permittivity

Complex permittivity of the material $\hat{\varepsilon }$ expressed in farads per meter [F/m] is a physical quantity that describes the reaction of a material to the applied electric field. The real part ε′ is a measure of a material’s ability to store electrical energy, i.e., to be polarized. The imaginary part ε″ corresponds to the effective losses in the material. The permittivity is often normalized to the permittivity of a vacuum (*ε*_0_), obtaining the so-called relative permittivity, denoted by the dimensionless complex quantity ${\hat{\varepsilon }}_{r}$ defined by its real and imaginary parts:

(1) $${\hat{\varepsilon }}_{r}=\frac{\hat{\varepsilon }}{\varepsilon_{0}}=\varepsilon_{r}^{\prime }-j\varepsilon_{r}^{\prime \prime }.$$

Therefore, the actual quantities measured in this paper are the real and the imaginary part of the relative permittivity: $\varepsilon_{r}^{\prime }$ and $\varepsilon_{r}^{\prime \prime }$, respectively. The losses described by the imaginary part of the permittivity arise from two different mechanisms of dissipation. The first mechanism of loss is due to the free charges, either electrons or ions, inside the material. The second loss mechanism is due to the dielectric polarization losses caused by the alternating electric field that continuously reorients dipoles. The resulting macroscopically measurable conductivity *σ* [S/m] thus encompasses both losses and is expressed as:

(2) $$\sigma =\omega_{0}\varepsilon_{r}^{\prime \prime }\;,$$

where ω is the angular frequency.

It is worth noting that in some references (e.g., in [21]), the term "permittivity" refers only to the real part of the complex relative permittivity, $\varepsilon_{r}^{\prime }$, while the losses are presented by conductivity *σ* rather than by the imaginary part of the complex relative permittivity, $\varepsilon_{r}^{\prime \prime }$. Nevertheless, as the permittivity is indeed a complex physical quantity defined above, in this paper, like in most physics textbooks, the term "permittivity" denotes the complex permittivity $\hat{\varepsilon }$, or, in the context of normalization to a dimensionless quantity, the term "permittivity" is equivalent to the complex relative permittivity ${\hat{\varepsilon }}_{r}$, and will be used accordingly throughout this paper.

Frequency dependence of a biological tissue permittivity is commonly expressed using the Cole–Cole empirical formula [22] which models the dispersion and absorption in lossy dielectrics:

(3) $${\hat{\varepsilon }}_{r}=\varepsilon_{\infty }+\overset{N}{\underset{i=1}{\sum }}\frac{\varepsilon_{s,\;i}-\varepsilon_{\infty }}{1+{\left(j\omega \tau_{i}\right)}^{1-\alpha_{i}}}+\frac{\sigma_{i}}{j\omega \varepsilon_{0}},$$

where $\varepsilon_{s}$ is the static dielectric constant, and $\varepsilon_{\infty }$ is the “infinite frequency” (i.e., the high frequency) dielectric constant. *N* is the number of Cole–Cole poles that model different relaxation regions, while *τ* is the associated relaxation time and *α* is a measure of the broadening of the dispersion. Each relaxation region is the manifestation of a polarization mechanism characterized by a different time constant. 

As the dominant relaxation mechanism in the frequency range of this study is the relaxation of the water molecules, the Cole–Cole model can be reduced to a single pole model:

(4) $${\hat{\varepsilon }}_{r}=\varepsilon_{\infty }+\frac{\varepsilon_{s}-\varepsilon_{\infty }}{1+{\left(j\omega \tau \right)}^{1-\alpha }}+\frac{\sigma }{j\omega \varepsilon_{0}},$$

where the parameters of the pole refer to the water relaxation. Accordingly, we derived the single pole Cole–Cole models to fit each set of the averaged measurement data, for each species and each tissue, as an additional representation of our measurement results. 

### 2.2. Measurement Method

The measurements were performed using the open-ended coaxial probe method, described in detail in [23]. An open-ended coaxial probe is a precisely machined segment of a straight rigid coaxial transmission line. The probe input port with its associated coaxial connector is on one end, leaving the other end open to be pressed against the material under test (MUT). The electric field lines are formed between the electrodes at the open end, closing dominantly through the MUT. The termination load at the end of the coaxial line is thus determined by the permittivity of the MUT. The reflection coefficient of the entire line terminated by the MUT can be measured at the probe input port by a vector network analyzer (VNA). VNA performs a one-port reflection measurement by sending the test signal into the probe input port, measuring the reflection coefficient *Γ*. The reflection coefficient *Γ* is a complex quantity defined by its real and imaginary part, i.e., by its amplitude and phase. MUT dielectric properties are extracted from the amplitude and the phase of the measured reflection coefficient.

We measured the permittivity using the slim form probe manufactured by Keysight Technologies (Santa Rosa, CA, USA) [23,24], having the outer diameter of 2.2 mm. Its operating frequency range is from 500 MHz to 50 GHz. As our measurements of the reflection coefficient were done with N9927A FieldFox handheld network analyzer (Keysight Technologies Inc.) [25], which has an upper frequency limit of 18 GHz, this study was performed from 500 MHz to 18 GHz. The slim form probe was selected as a common choice for tissue measurements [26] due to its small diameter, which helps to distinguish different tissues in a heterogeneous sample. As a usual practice when measuring biological tissues, the probe open end was in all cases pressed against the sample with its entire surface. The declared accuracy of the probe for both $\varepsilon_{r}^{\prime }$ and $\varepsilon_{r}^{\prime \prime }$ is ±10%.

To extract the permittivity of a material from the measured reflection coefficient, postprocessing calculations have to be made. For the slim form probe, these calculations are incorporated in the software Keysight Materials Measurement Suite N1500A [27] provided by the probe manufacturer, accompanying the probe. The calculation method is based on solving the equivalent model of the probe termination load admittance, which includes all electromagnetic phenomena occurring in the probe and MUT. Thus, in order to determine the parameters of the load model, the measurement must first be calibrated using three known loads: open (without any MUT, open end is in the air), short (open end is shorted by a conductive sheet), and a known liquid (water being most commonly used, as its dielectric dispersion is well-known). After a proper calibration, measurements on MUTs can be made.

### 2.3. Materials under Test

In this study, three types of brain tissues from different species were measured, namely: white matter and grey matter (both from cerebrum), and cerebellum. Although cerebellum also consists of white and grey matter, it has been treated here as a single tissue, which is a usual approach in the literature [20,21], considering the fact that white and grey matter are so intertwined in cerebellum that they are indistinguishable by the measuring probe. 

**Figure 1:**
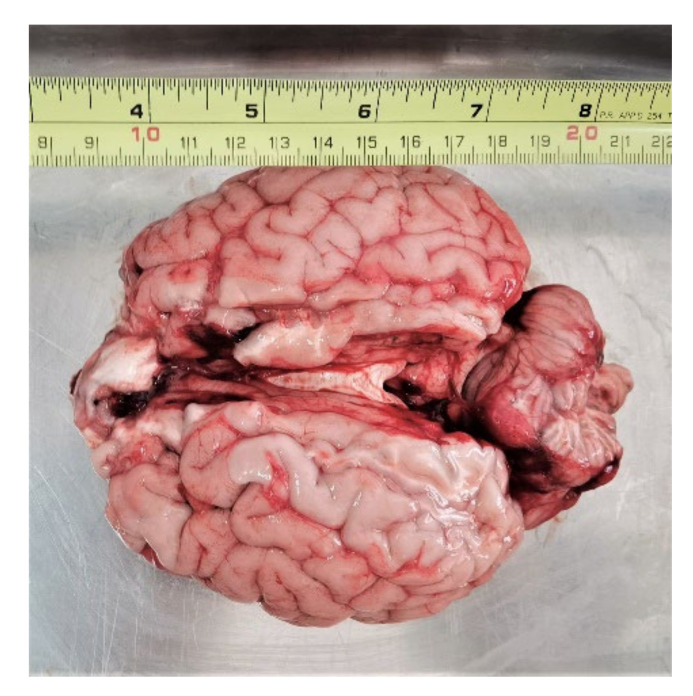
Bovine brain B1.

The main challenge when measuring dielectric properties of brain tissues was obtaining freshly excised brains so that the natural deterioration of postmortem tissues, as well as the dehydration, would affect the results as minimally as possible. In total, we acquired two different human brains (labeled hereafter as H1 and H2), three different bovine brains (labeled hereafter as B1, B2 and B3), and one porcine brain (labeled hereafter as P). Cerebellum was intact only in both human brain specimens (H1 and H2) and in one bovine brain specimen (B1). Other animal brain specimens were split into two hemispheres during slaughter, which was especially damaging to the delicate cerebellum tissue located in the center so the measurements on cerebellum could not be performed. Example of the brain sample (B1) is shown in Figure 1 (measuring tape shown for scale).

Human tissues were obtained from hospital autopsies at the Department of Pathology and Cytology, University Hospital Centre Zagreb. The study was performed under ethical approval from the Ethics Committee of the University Hospital Centre Zagreb. 

Human brain H1 was from a male patient 50 years of age. Human brain H2 was from a female patient 84 years of age. The measurements were performed immediately after the autopsy (day 1), which was performed two days postmortem. Prior to autopsy, the bodies were kept in the refrigerator at 4 °C. The exact time between the death and the refrigeration at the pathology department is unknown, but a minimum of four hours is dictated by medical protocol. It is important to note that the brains were intact in the skull from the time of death to the day of autopsy. That is why the span of two days from death to measurements did not result in the dehydration of the brains. Neither of the patients had any disclosed brain pathologies noted in their medical record; however, both suffered from other pathologies that eventually led to lethal outcome, and were thus treated pharmaceutically prior to death. The human brain samples were either in the form of brain slices of around 1.5-centimeter thickness, exposing both white and grey matter, or in the form of ca. 2 cm × 2 cm × 2 cm cuboids consisting dominantly of white matter. 

In addition to the measurements on day 1, the white and grey matter samples of brain H1 were measured on day 2 and day 3 from autopsy (four and five days postmortem) to examine the effects of the excised tissue degradation. The excised samples were kept in the refrigerator at 4 °C in sealed containers between the measurements. The samples were heated from 4 °C to 25 °C naturally, keeping them in sealed containers to avoid dehydration, by achieving thermal equilibrium with the room temperature, which was kept at 25 °C.

The age of the porcine specimen was not known, while all bovine specimens were under 12 months of age. Brains were obtained from the local butcher between 18 and 36 h from animal slaughter, depending on the brain. There was no dehydration or decomposition noticeable, presumably due to the short timespan from the slaughter and the fact that the brains were not dissected until we obtained them, while in the meantime they were kept wrapped and refrigerated at 1 °C. Before the measurements, we dissected all animal brains into coronal slices ca. 1.5 cm thick. 

### 2.4. Measurement Setup

The slim form probe by Keysight Technologies Inc. was connected to the vector network analyzer FieldFox N9927A (Keysight Technologies Inc.) by a phase-stable coaxial cable Sucoflex 404 (HUBER+SUHNER AG, Herisau, Switzerland). The VNA was connected to a computer, and the measurements were controlled and performed using the manufacturer-provided software Keysight Materials Measurement Suite N1500A [27]. The measurements were performed from 500 MHz to 18 GHz with a 20 MHz step. During the measurements of human brains, the sweeping average was enabled on the VNA with averaging factor set to 10, meaning that the VNA automatically measured each measurement point consecutively 10 times, and then the average of those 10 measurements was calculated by the VNA for each measurement point. This procedure removes stochastic noise and obtains more accurate measurements. Unlike the animal brains which were measured in our lab, the human brains were measured out of our lab in a less controlled setting, which is why we made the choice of averaging to compensate for any possible instability of the measurement setup. VNA averaging was not performed with the animal samples, where more points were measured on each sample.

**Figure 2:**
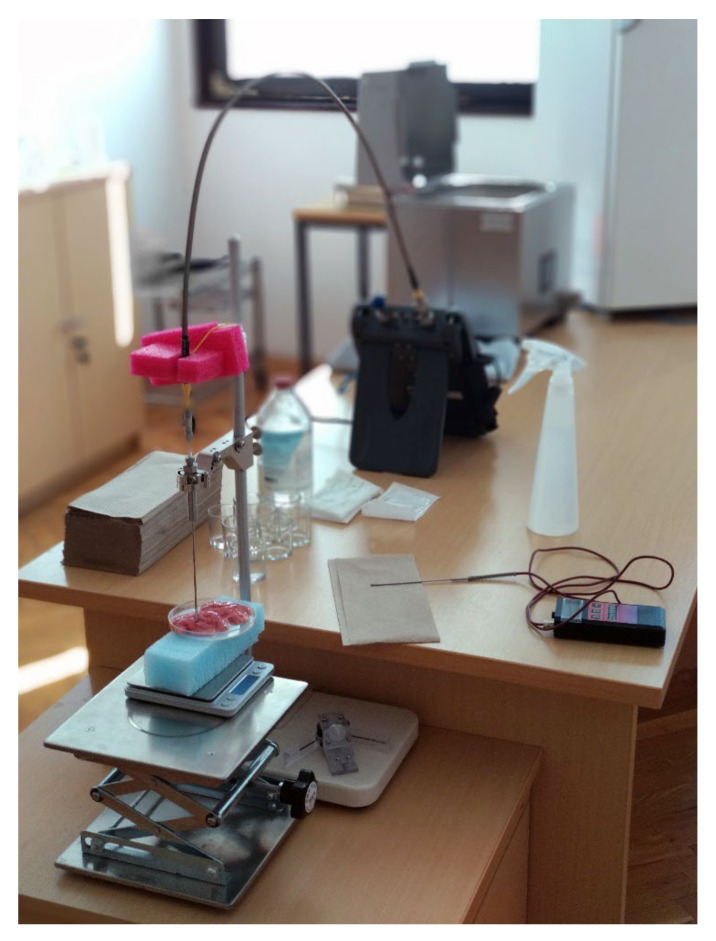
Measurement setup.

The probe was fixed to its stand in the vertical position and pressed onto the measured tissue. The samples were contained in a plastic dish on top of a polystyrene foam block that separated the dish with the sample from the metal laboratory jack underneath. The laboratory jack was used to lift the sample to the open end of the probe, so that the probe was tightly pressed against the sample to avoid air gaps between the probe and the sample. The walls of the dish as well as the laboratory jack underneath did not affect the measurement results. This was confirmed by several pilot measurements where the probe was put directly above the supporting structure without the biological sample. The measured permittivity of that setup was compared to the measurement of the open probe that was left in air, and no difference was found. The described measurement setup is shown in Figure 2. 

The measurements were performed on homogeneous regions of either white or grey matter, when such regions were clearly visible on the sample. The regions with visually indistinguishable white and grey matter were avoided. Within these constraints, each sample was measured at as many points as possible to obtain better accuracy. The number of measurement points per tissue is shown in Table 1. 

The number of measurement points reported in Table 1 corresponds to the measurements performed on different measurement points. These numbers were used to calculate the standard deviation for each sample.

Between measuring different samples, the probe was cleaned with 70% ethyl alcohol to prevent cross contamination and ensure the best results. The setup was also recalibrated in regular intervals with the refresh calibration option available in Keysight Materials Measurement Suite N1500A [27], where one calibration standard out of the three is chosen for recalibration. In our case, we chose to recalibrate with water as its properties gave the most stable results during the measurement process and, therefore, it was the most appropriate for recalibration. The room, water, and MUT temperatures were all kept at 25 °C during all measurements, while the temperature was controlled using a precise thermometer DTM3000 (LKM Electronics GmbH, Geratal, Germany).

## 3. Results

### 3.1. Human Brain Permittivity

**Figure 3:**
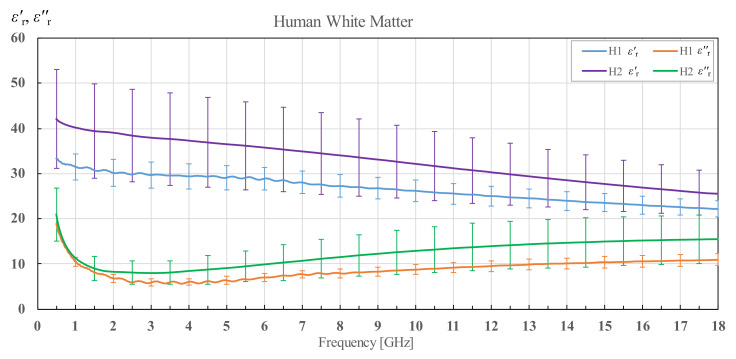
Permittivity of human white matter for brains H1 and H2 with corresponding standard deviations.

**Figure 4:**
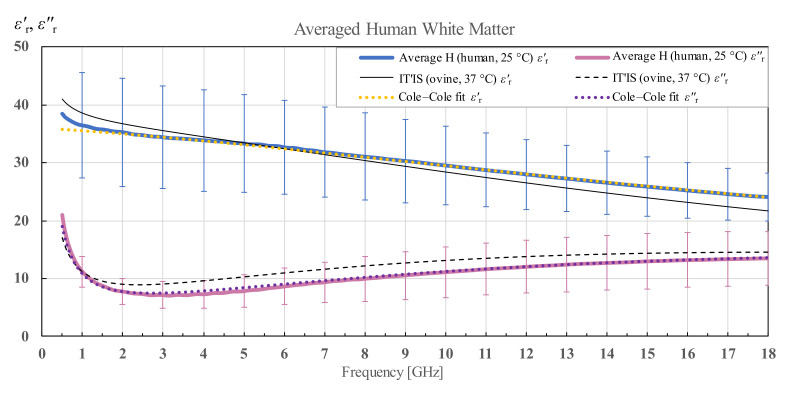
Average permittivity of human white matter with standard deviation and Cole–Cole fit, shown in comparison with the data from IT’IS database [21].

**Figure 5:**
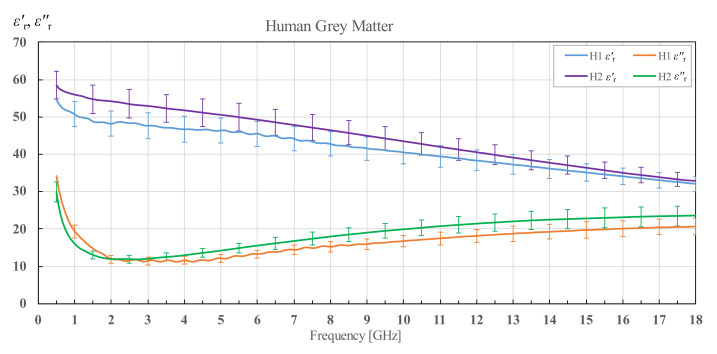
Permittivity of human grey matter for brains H1 and H2 with corresponding standard deviations.

**Figure 6:**
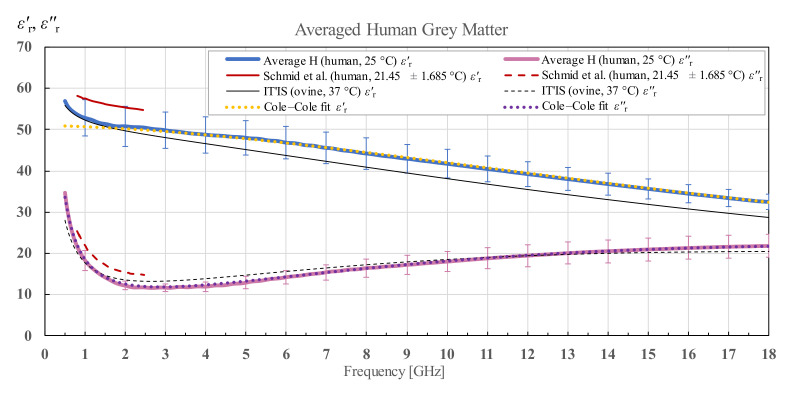
Average permittivity of human grey matter with standard deviation and Cole–Cole fit, shown in comparison with the data from IT’IS database [21] and Schmid et al. [28].

**Figure 7:**
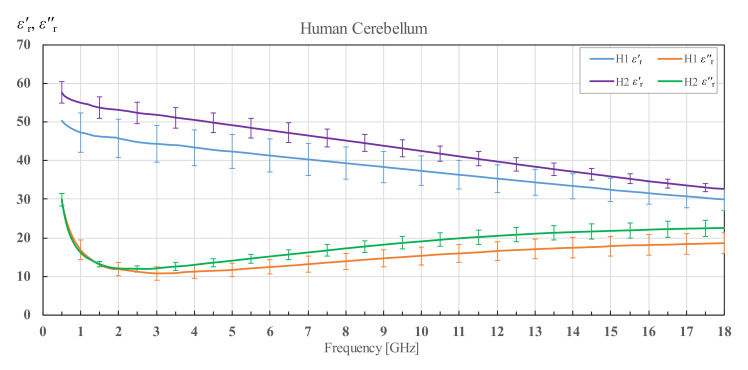
Permittivity of human cerebellum for brains H1 and H2 with corresponding standard deviations.

**Figure 8:**
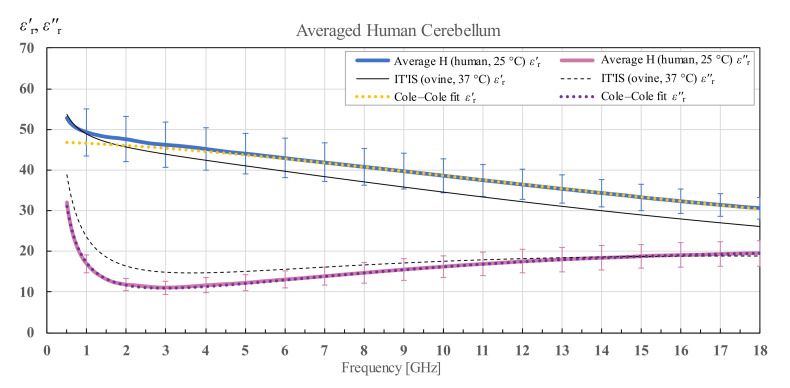
Average permittivity of human cerebellum with standard deviation and Cole–Cole fit, shown in comparison with the data from IT’IS database [21].

**Figure 9:**
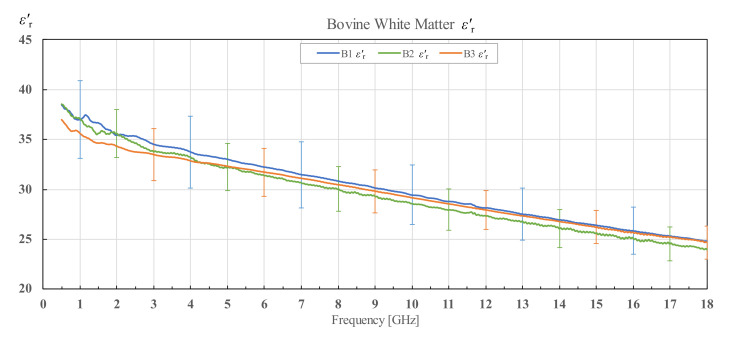
Real part of permittivity of bovine white matter for brains B1, B2 and B3 with associated standard deviations.

**Figure 10:**
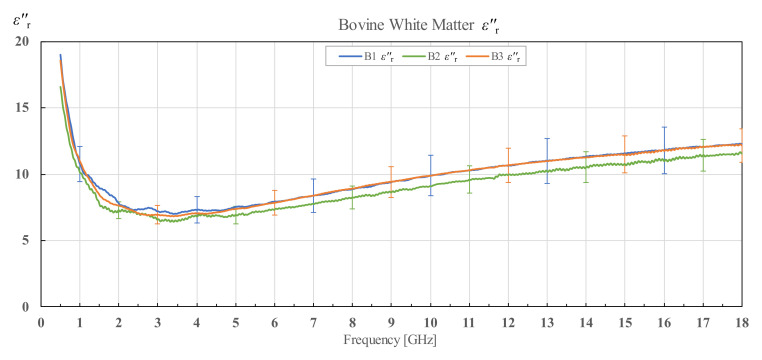
Imaginary part of permittivity of bovine white matter for brains B1, B2 and B3 with associated standard deviations.

**Figure 11:**
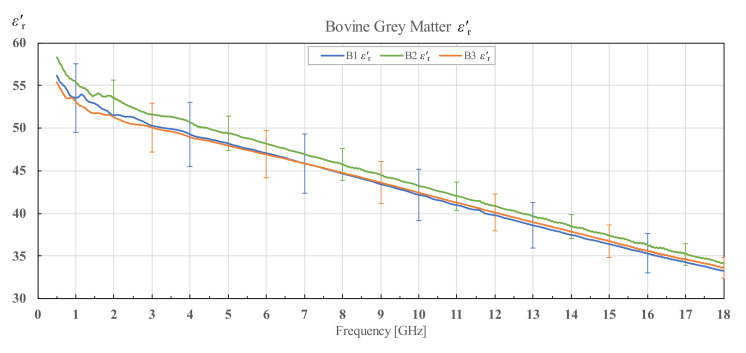
Real part of permittivity of bovine grey matter for brains B1, B2 and B3 with associated standard deviations.

**Figure 12:**
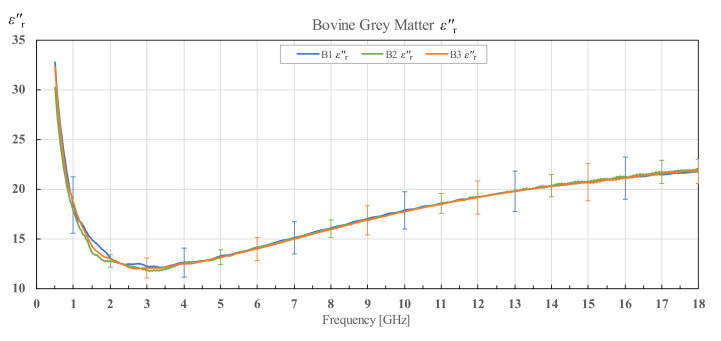
Imaginary part of permittivity of bovine grey matter for brains B1, B2 and B3 with associated standard deviations.

**Figure 13:**
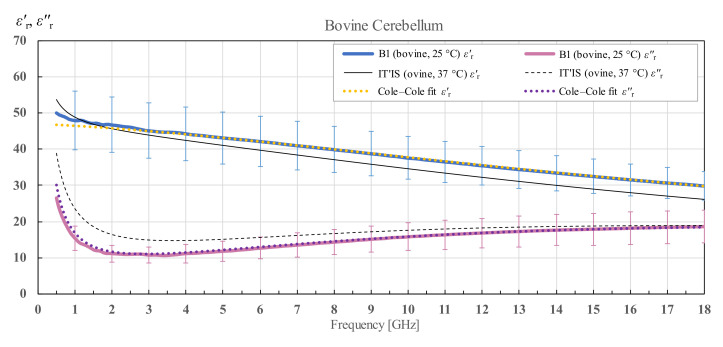
Permittivity of bovine cerebellum for brain B1 with standard deviation and Cole–Cole fit, shown in comparison with the data from IT’IS database [21].

**Figure 14:**
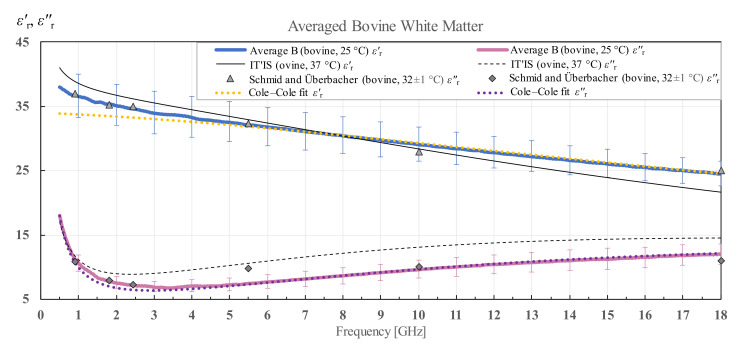
Average permittivity of bovine white matter with standard deviation and Cole–Cole fit, in comparison with the data from IT’IS database [21] and Schmid and Überbacher [29].

**Figure 15:**
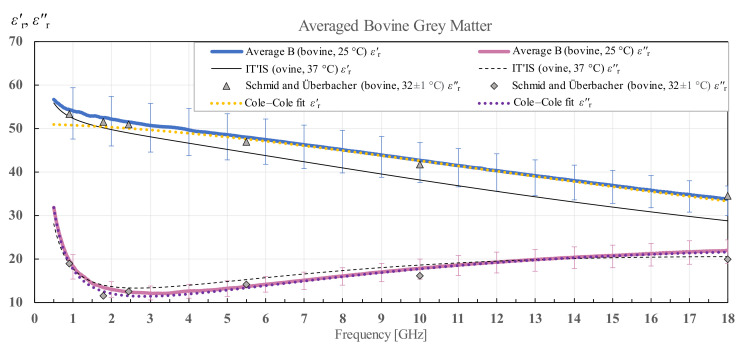
Permittivity of averaged bovine grey matter with standard deviation and Cole–Cole fit, in comparison with the data from IT’IS database [21] and Schmid and Überbacher [29].

**Figure 16:**
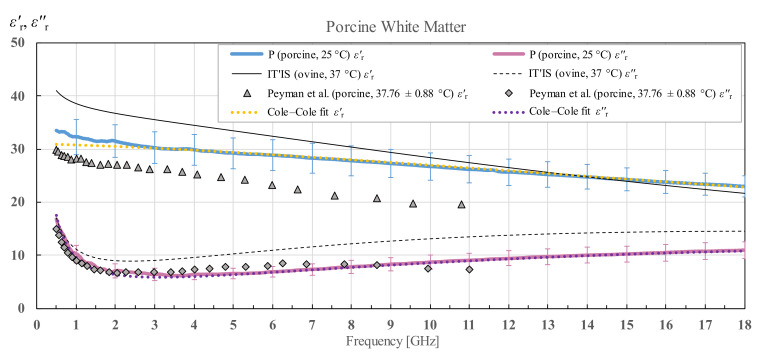
Permittivity of porcine white matter (P) with standard deviation and Cole–Cole fit, shown in comparison with the data from IT’IS database [21] and Peyman et al. [30].

**Figure 17:**
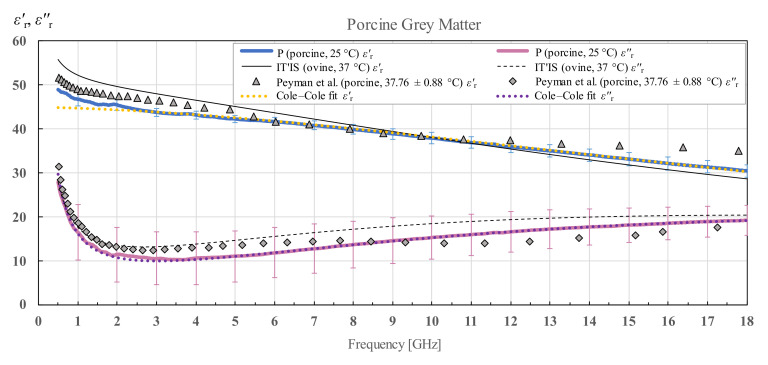
Permittivity of porcine grey matter (P) with standard deviation and Cole–Cole fit, shown in comparison with the data from IT’IS database [21] and Peyman et al. [30].

In Figure 3, Figure 4, Figure 5, Figure 6, Figure 7, Figure 8, Figure 9, Figure 10, Figure 11, Figure 12, Figure 13, Figure 14, Figure 15, Figure 16 and Figure 17, the colored lines represent the average of all measurements performed on a tissue, and the vertical bars show the associated standard deviation. The standard deviation is shown only at discrete frequency points for the sake of visual clarity. The colored lines showing the measurement results were additionally smoothed with a moving average spanning ±150 MHz. The smoothing was done to eliminate the small fluctuations that do not reflect the actual fluctuations of permittivity but are caused by the imperfections of the measurement setup. This smoothing procedure was performed on all the measured results in this study. Permittivity of human white matter for brains H1 and H2 is shown in Figure 3 in terms of its real and imaginary part, i.e., $\varepsilon_{r}^{\prime }$ and $\varepsilon_{r}^{\prime \prime }$. The brain of the older patient (H2) displays significantly higher permittivity in both the real and imaginary parts. It is also noticeable that the standard deviation of H2 brain is much higher. 

Additionally, Figure 4 shows the averaged results of white matter for human brains H1 and H2 with the associated overall standard deviation for human white matter measurements and Cole–Cole fit of the measured data according to (4). The results are compared to the permittivity data from the IT’IS database [21], shown in black for reference. 

Figure 5 shows the permittivity of grey matter of brains H1 and H2. Again, the brain H2 has overall larger permittivity over the whole frequency range, but the difference is less prominent between the two brains for both the real and imaginary parts of the permittivity. Additionally, this time, the standard deviation is comparable between H1 and H2. 

Figure 6 shows the averaged results of grey matter for human brains H1 and H2 with the associated standard deviation and Cole–Cole fit of the measured data according to (4) and the IT’IS data shown for reference. For the human grey matter, there is another suitable reference for comparison shown also on the chart, by Schmid et al. [28], who measured the human grey matter permittivity in a narrow frequency range from 800 MHz to 2450 MHz.

The results for cerebellum of both brain H1 and H2 are shown in Figure 7. The difference in cerebellum permittivity of the two brains is considerable. The results for human brain H1 are lower in permittivity for both the real and imaginary parts from brain H2. However, now the standard deviation of the averaged measurement results is larger for the brain H1, which is in contrast to the results for white matter. 

Figure 8 shows the averaged cerebellum of both human brains along with the corresponding standard deviation and Cole–Cole fit of the measured data according to (4). The IT’IS data are displayed in black for comparison.

### 3.2. Bovine Brain Permittivity

The results for $\varepsilon_{r}\prime$ and $\varepsilon_{r}\prime \prime$ of three different bovine brains (B1, B2 and B3), along with their corresponding standard deviations, are shown in Figure 9 and Figure 10 for white matter, and Figure 11 and Figure 12 for grey matter. The results for white and grey matter for brain B2 and B3 follow similar trendlines as the results for brain B1. Overall, all three measured brains have similar permittivity even though they are from different specimens. 

The measurement results for cerebellum tissue of brain B1 are shown in Figure 13. It is immediately noticeable that the measured data for cerebellum have a much larger standard deviation than the measured data for white and grey matter. It is because cerebellum is composed of both white and grey matter which are tightly intertwined, so that white and grey matter in cerebellum cannot be discriminated by the probe. However, due to this heterogeneity, the measured results varied greatly from point to point. However, all three tissues follow the general trends of the previously reported literature data [21,29].

The averaged results and Cole–Cole fit of the measured data according to (4) for white and grey matter for all measurement points of the three bovine brains along with the standard deviation calculated for all samples are shown in Figure 14 and Figure 15. IT’IS data [21] are shown in black for reference. For the bovine white and grey matter, there is a suitable reference for comparison shown also on the chart, by Schmid and Überbacher [29], who measured the bovine white and grey matter permittivity only at a few discrete frequency points shown in grey markers.

### 3.3. Porcine Brain Permittivity

The measured porcine brain white and grey matter permittivity and a Cole–Cole fit of the measured data according to (4) are shown in Figure 16 and Figure 17 respectively. IT’IS data [21] is shown in black for reference. For the porcine white and grey matter, there is a suitable reference for comparison shown also on the chart, by Peyman [30], who measured the porcine white and grey matter permittivity in the similar frequency range, shown in grey markers.

We can notice that the grey matter has a substantially larger standard deviation of $\varepsilon_{r}^{\prime \prime }$ than $\varepsilon_{r}^{\prime }$, especially when taking into account the relative value of standard deviation with respect to the magnitude of $\varepsilon_{r}^{\prime \prime }$. 

### 3.4. Interspecies Variability in Permittivity of White and Grey Matter and Cerebellum

**Figure 18:**
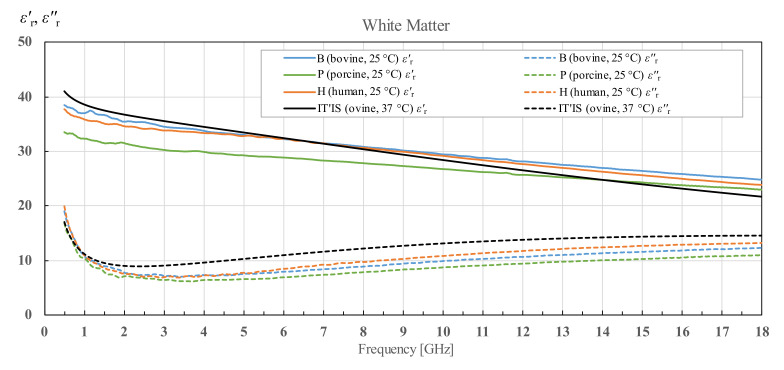
White matter permittivity comparison of averaged bovine brains (B), averaged human brains (H) and a porcine brain (P), as well as comparison with the published data from IT’IS database [21].

**Figure 19:**
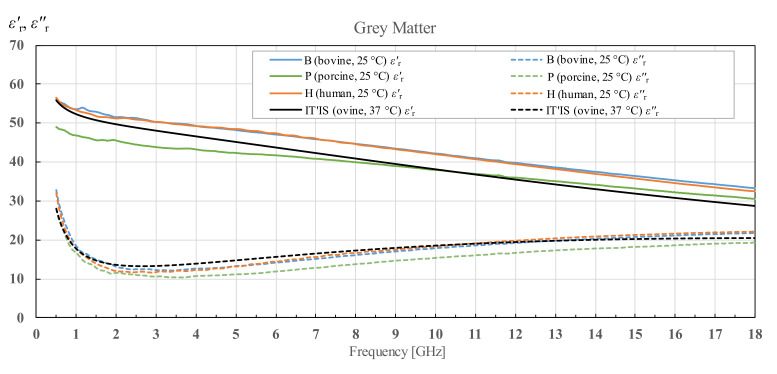
Grey matter permittivity comparison of averaged bovine brains (B), averaged human brains (H) and a porcine brain (P) as well as comparison with the published data from IT’IS database [21].

**Figure 20:**
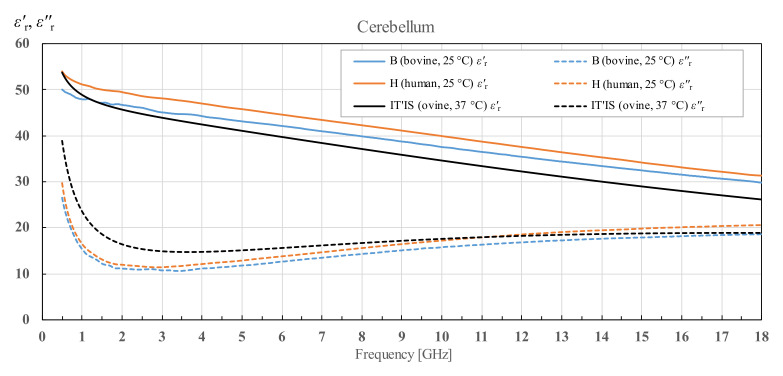
Cerebellum permittivity comparison of bovine brain (B1) and averaged human brains (H), as well as comparison with the published data from IT’IS database [21].

Lastly, it is interesting to see comparative results for white and grey matter of all three species, as well as to compare human and bovine cerebellum data. Permittivity results of three different bovine brain tissues as well as two human brain tissues are averaged and only the average is shown in Figure 18, Figure 19 and Figure 20. Averaged bovine brains are simply labeled as B and averaged human tissues as H. Comparison of the white matter for all three species is shown in Figure 18. Even though the three species show some variability in both the real and imaginary parts of complex permittivity, they show a similar sloping trend.

Results for grey matter for all three species are shown in Figure 19. Similar to the results for white matter, averaged permittivity of bovine brains matches the averaged permittivity of human brains. Again, porcine brain measurements differ from the aforementioned two. However, the sloping trend is similar for all three species.

Lastly, the permittivity of cerebellum is shown in Figure 20. As described in Section 2.3, cerebellum was measured only for brains H1, H2 and B1. The results for H1 and H2 are averaged and displayed as H, while the results for one bovine brain are displayed as B1. 

### 3.5. Human White and Grey Matter Permittivity on Day 2 and Day 3

For permittivity of H1 measured on day 2 and day 3, we calculated the percent change in permittivity relative to the permittivity measured on day 1 using Equations (5) and (6) for both white and grey matter separately.

(5) $$\Delta \varepsilon_{r\left(Day\;2\;or\;Day\;3\right)}^{\prime }\;\left[\%\right]=\;\frac{\varepsilon_{r\left(Day\;2\;or\;Day\;3\right)}^{\prime }-\varepsilon_{r\left(Day\;1\right)}^{\prime }}{\varepsilon_{r\left(Day\;1\right)}^{\prime }}\cdot 100\%,$$

(6) $$\Delta \varepsilon_{r\left(\mathrm{Day}\;2\;\mathrm{or}\;\mathrm{Day}\;3\right)}^{\prime \prime }\;\left[\%\right]=\frac{\varepsilon_{r\left(\mathrm{Day}\;2\;\mathrm{or}\;\mathrm{Day}\;3\right)}^{\prime \prime }-\varepsilon_{r\left(\mathrm{Day}\;1\right)}^{\prime \prime }}{\varepsilon_{r\left(\mathrm{Day}\;1\right)}^{\prime \prime }}\cdot 100\%$$

**Figure 21:**
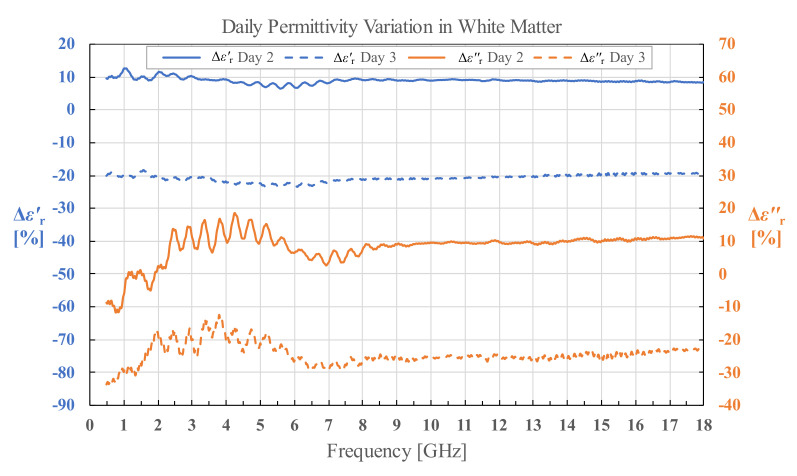
Permittivity change in human white matter for brain H1 on day 2 and day 3.

**Figure 22:**
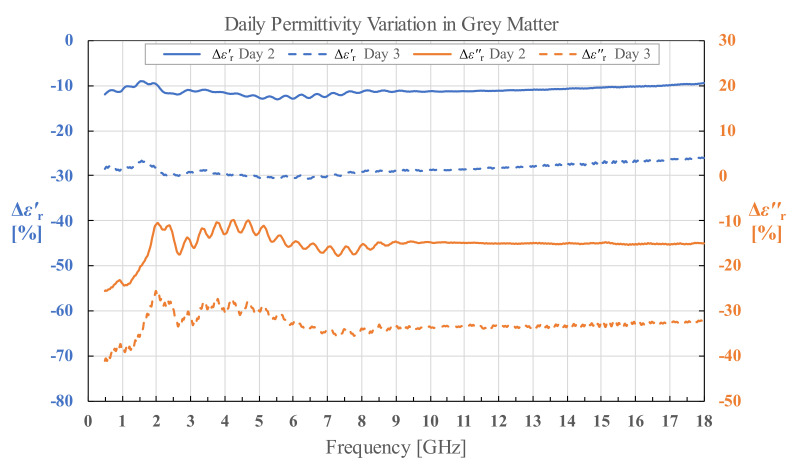
Permittivity change in human grey matter for brain H1 on day 2 and day 3.

The calculated results as a function of frequency are shown in Figure 21 and Figure 22. The fluctuations in the results visible under 8 GHz, and especially prominent for $\varepsilon_{r}^{\prime \prime }$, are due to the imperfections of the measurement setup. Besides the aforementioned fluctuations, it is worth noting that the difference in permittivity is fairly consistent throughout the measured frequency range. The percent change averaged across the measured frequency range is shown in Table 2. 

### 3.6. Cole–Cole Models of the Data

The averaged measured permittivity for each species and each tissue was additionally depicted as a single pole Cole–Cole model in Figure 4, Figure 6, Figure 8, Figure 13, Figure 14, Figure 15, Figure 16 and Figure 17. The Cole-Cole fits were derived in Microsoft Excel using Equation (4), producing the best fits predominantly based on the least-squares, with additional manual adjustments for a better fit at the highest measured frequencies. The derived parameters are listed in Table 3.

### 3.7. Supplementary Materials

The numerical values of the measured permittivity presented in Section 3.1, Section 3.2, Section 3.3, Section 3.4 and Section 3.5 are made available in the form of downloadable Excel tables, as Supplementary Materials accompanying this paper, as follows:

- human white matter, grey matter and cerebellum permittivity for brains H1 and H2, with the average permittivity of human white matter, grey matter and cerebellum;
- bovine white matter and grey matter permittivity for brains B1, B2 and B3, and cerebellum permittivity for brain B1, with the average permittivity of bovine white matter and grey matter;
- porcine white matter and grey matter permittivity;
- human white matter permittivity for brain H1 measured along the course of three days;
- Cole–Cole models for the average permittivity of each tissue.

## 4. Discussion

### 4.1. Results Comparison with the Published Literature

#### 4.1.1. General Remarks

Our measurement results were primarily compared with the IT’IS database [21], as it serves as the most comprehensive published reference on tissue permittivity and covers our whole measurement frequency range. The IT’IS data on white matter, grey matter and cerebellum permittivity originate from Gabriel [20]. The values for brain tissues in the frequency range from 500 MHz to 18 GHz in Gabriel [20] were compiled from the literature already published at that time (i.e., before 1996):

- rabbit specimen at 37 °C [31] (white and grey matter),
- mouse specimen at 37 °C [32] (grey matter),
- rat specimen in vivo at 32 °C [33] (grey matter),
- feline specimen in vivo at 36 °C [33] (grey matter),
- canine specimen in situ at 36 °C [34] (white and grey matter),
- canine specimen at 20 °C [35] (white and grey matter),
- feline specimen in vivo at 33 °C [36] (white and grey matter),
- canine specimen at 37 °C [37] (white and grey matter),

as well as from her own measurement results.

In study [20], Gabriel reported her own measurement results for ovine white matter, grey matter and cerebellum measured at 37 °C, as well as for human white matter and grey matter obtained from autopsies and measured also at 37 °C. While the ovine tissue results are reported in detail as comprehensive sets of measurement results measured in a continuous frequency range from 10 Hz to 20 GHz, the human tissues are reported only for a several frequency points. The final output of the Gabriel study [20] is a set of Cole-Cole models, i.e., the mathematical equations representing best fits for each tissue. These models are the basis for the IT’IS data. Gabriel states in [20] that the parameters of each model were adjusted to correspond to a close fit between the model and the most comprehensive data set available for the particular tissue. Observing the models for the white matter, grey matter and cerebellum in [20], they are mostly based on the ovine results, which are the most comprehensive data set for these particular tissues.

Accordingly, the difference between our results and IT’IS database could partly be explained by the difference in species. Additionally, the results compiled in [20] were all published for temperatures exceeding 30 °C with the exception of canine brain measurements from [35], which were done at 20 °C. Higher temperatures in general result in a lower real part of relative permittivity of biological tissues over the whole frequency range as well as increase of conductivity, i.e., rhe imaginary part of the permittivity [38]. Our results for $\varepsilon_{r}\prime$ are higher than the IT’IS data for H1 and H2 grey matter and cerebellum, H2 white matter, and grey matter for all three bovine brains. White matter from all three bovine brains as well as both white and grey matter from the porcine brain show lower permittivity than the IT’IS data up to some crossover frequency, after which they display higher permittivity. Similar occurrence is visible for H1 white matter, but as the crossover frequency is over 16 GHz, in most of the measured frequency range the permittivity is lower than the reported values from IT’IS. When it comes to the imaginary part of the relative permittivity, the results are systematically lower than the reported values from IT’IS for: H1 white and grey matter and cerebellum, all three bovine white matter measurements, B1 cerebellum and porcine white and grey matter. Other tissues again display crossover frequency below which the imaginary part of the permittivity is lower than the reported data. Those tissues are: H2 white and grey matter and cerebellum, and all three bovine grey matter measurements.

We are aware that our measured data is compared here with the data for different species at different temperatures. Hence, the comparison, although indicative, must be taken with some reservation. However, there are not enough published data on permittivity of brain tissues of various species measured at various temperatures in a relevant and continuous frequency range to make a direct comparison to a corresponding set of data. In general, the published data for all three measured tissues in this study are considerably dated and scarce. It is especially true for human tissues where, to the best of our knowledge, there are no reported data on human white matter, grey matter and cerebellum measured in the continuous frequency range of this study. 

#### 4.1.2. Human Tissues 

Sparse data for human white and grey matter are presented in [20] in a graphical form; unfortunately, the logarithmic scale of the graphics is such that it is impossible to precisely extract those values, thus they cannot serve for comparison. Clearly, those values were not even meant to serve such a purpose, as the final output of [20] were the mathematical models described by Cole-Cole equations (and referenced later by IT’IS). We also compared our measured data with the usable data for human grey matter in the narrow frequency range from 800 MHz to 2450 MHz published in [28]. Our measurement results follow a similar trendline, but with a downward shift for both the real and imaginary parts of the permittivity. The reported temperature at the measurement points in [28] was from 18 °C to 25 °C with the mean of 21.35 °C, which is lower compared to our measurement temperature of 25 °C. The difference in permittivity could be explained by the difference in temperature as well as the difference in the measurement protocol. Measurements in [28] were performed over arachnoid membrane, which could result in higher measured permittivity values.

#### 4.1.3. Bovine Tissues

Bovine white and grey matter was additionally compared with the reported results from Schmid and Überbacher [29], which reported the data at seven discrete frequency points, out of which six overlap with our measured frequency range (0.9 GHz, 1.8 GHz, 2.45 GHz, 5.5 GHz, 10 GHz and 18 GHz). The excised brain tissues were of either an adult animal or a young calf. Our measurement results are in better agreement with the results for young calves. This was to be expected as all three calves from our study were under 12 months of age, and thus better suit the reported results for young animals. The agreement between the data is satisfactory, especially when considering that their white and grey matter was measured at 32 °C.

#### 4.1.4. Porcine Tissues

Porcine white and grey matter was additionally compared with the results published in [30]. The results from [30] were measured from 50 MHz to 20 GHz in vivo for pigs weighing around 50 kg and ex vivo for three different weight categories of pigs (10, 50 and 250 kg), and the tissues were measured as close to 37 °C as possible. Although we did not know the age of our specimen, we could conclude by the size of the brain that it was not a young porcine specimen. That is why we compared our results with the results for 250 kg pigs. Our results for white matter exhibit a higher real part of the permittivity, which is to be expected with the difference in tissue temperature, while the results for the imaginary part are lower up to 9 GHz, after which they are slightly higher than reported in [30]. A very similar trend for the imaginary part is noticeable for the results for grey matter where the crossover frequency is again around 9 GHz. The real part of the permittivity of grey matter measured in [30] is comparable to ours.

### 4.2. Interspecies Variability

There is a noticeable interspecies variability for all three measured brain tissues. The averaged results for the real part of the permittivity of bovine brain white and grey matter are similar to the averaged results for human brain white and grey matter. The imaginary part of the permittivity shows greater difference, but the results are still comparable. Both human and bovine white and grey matter show greater deviation from the porcine brain white and grey matter than from each other. The permittivity data for cerebellum are only available for human and bovine brains and they display noticeable difference between bovine and human cerebellum in the both real and imaginary parts of the permittivity. It should be remarked that the permittivity trends, with respect to frequency, are similar for all three measured species when comparing the same brain tissue.

### 4.3. Tissue Degradation over Time

Regarding the effects of tissue degradation over time in the stored samples, measured on H1 brain on day 2 and day 3, there is a clear trend of increase in magnitude of average change in permittivity from day 2 to day 3 in all measured tissues for both $\varepsilon_{r}^{\prime }$ and $\varepsilon_{r}^{\prime \prime }.$ The results confirm that there is an increase in the tissue degradation over time. Observing the results for grey matter in Table 1, the decrease in $\varepsilon_{r}^{\prime }$ and $\varepsilon_{r}^{\prime \prime }$ (which correlates with the decrease in conductivity *σ*) for both day 2 and day 3 could be explained by the loss of water in the tissue, i.e., dehydration. The effect of water content on permittivity and conductivity of brain tissues was modeled in [39]. The same process of dehydration is an explanation for the results for white matter on day 3. The only exception are the results for white matter on day 2, where the permittivity increased with respect to the measured results on the fresh samples on day 1. The average standard deviation of the white matter measurements was not noticeably higher on day 2 to justify such a result. Unfortunately, this anomaly remained unnoticed at the time of measurements, i.e., until the results were processed. Thus, we can only speculate post festum that there was an unnoticed systematic error in this set of measurements, which resulted in a drift of the results; possibly due to an inadequately performed probe calibration. However, it must again be noted that the change in white matter permittivity measured on day 3 suggests that the decay of white matter increased with time, just like for the grey matter. 

### 4.4. Intraspecies Variability

All three bovine brains show similar permittivity values for both grey and white matter, which is to be expected as they all originate from healthy specimens of similar age. Although the intraspecies variability between bovine grey and white matter is negligible, the human brain results suggest different conclusions. There is an apparent difference in all three measured tissues (grey and white matter and cerebellum), where the results for H2 are consistently higher than for H1 for both the real and imaginary parts of the permittivity. 

#### 4.4.1. Age-Related Factors

The most obvious difference between H1 and H2 is their age, although not necessarily the most important one. To the best of our knowledge, the only studies trying to directly link the dielectric properties to the age of the specimen are the studies [29,30,40]. However, all of them compare the difference between the young (immature) and the adult (fully matured) brain. On the other hand, our human brains were both fully matured, but one belonged to a middle-aged adult, while the other belonged to a senior adult. Therefore, the studies [29,30,40] cannot be confidently used in this context.

As there are no other appropriate studies done on age-related permittivity changes of brain tissues, we searched for studies researching the water content of brain tissues in older adults. The rationale for this is the known fact that the permittivity of biological tissues, in this frequency range, is dominantly determined by their water content. The higher the water content of a tissue, the higher permittivity it has, as demonstrated in [39]; as water has the largest permittivity value compared to any other tissue constituent. That is also why grey matter has overall larger permittivity and conductivity than white matter, as its water content is around 83%, compared to the 69% water content found in white matter [39]. Accordingly, our results suggest that all three brain tissues taken from brain H2 had a higher water content than those taken from H1. The study by Gullet et al. [41] showed that the extracellular free water within white matter increases with normal aging and can even be used as an indicator of cognitive decline. Free water refers to the water molecules that are freely diffusing without being hindered or restricted by tissue membranes. Increases in free water are connected with accumulation of extracellular water, which may occur due to processes such as atrophy, edema, or neurodegenerative disease. The increase in free water causes a decrease in white matter integrity [41]. Additionally, free water has a larger permittivity than bound water as bound water exhibits a permittivity value closer to that of dry matter, as reported in [42]. Thus, a reasonable explanation for the difference in permittivity of brains H1 and H2 could be in the changes that were ongoing in the elderly brain, causing it to have an increase in extracellular free water, especially because the biological tissue permittivity in the gigahertz frequency range is mostly influenced by the polarization of free water molecules [43].

#### 4.4.2. Medical Condition of the Patients

Searching for potential causes of the difference in permittivity of brains H1 and H2, we paid additional attention to the medical condition of the deceased patients. Human brain H1 was from a male patient 50 years of age who died from dissection of abdominal aorta. Prior to his death, during hospitalization, the patient had acute renal insufficiency and hemodynamic instability, and was treated by means of intensive care therapy that included intubation and mechanical ventilation coupled with vasoactive therapy and intravascular volume replacement with 0.9% NaCl (saline) and colloid solutions. The presence of the aforementioned pathology, combined with the therapy used, could have easily caused the body fluid disbalance (oedema or dehydration) [44,45,46], thus changing the water content in the brain tissue. In this particular case the patient suffered from obvious loss of fluid that led to kidney failure. Also, an important factor to mention is that this patient had an MRI scan of the brain done during hospitalization that demonstrated combined ischemia and oedema of the brain stem (one of the brain parts most vulnerable to hypoxia; however, not the subject of analysis regarding its dielectric properties in this study) as a result of brain hypoperfusion hypoxic-ischemic injury [47].

Human brain H2 was from a female patient 84 years of age, that died from pulmonary oedema and had all the elements of hypotensive shock. One should note that in pulmonary oedema, as in this particular case, the patient had tachypnoea (very high number of inspirations/expirations in one minute). It is well known that tachypnoea leads to a lower amount of carbon dioxide (CO_2_) in blood which then causes vasoconstriction of brain vessels and results in a reduced amount of cerebral blood volume, and thus reduces the brain water content [48]. This patient also received intensive care therapy measures because of hemodynamic instability, although to a lesser extent than the previously described patient due to the more rapid progression of disease that resulted in a lethal outcome.

The brain tissues H1 and H2 didn’t show any macroscopic abnormalities, other than the visible atrophic changes on H2, however, within expectations according to the age of the patient. Also, the deceased patients did not have any disclosed brain pathologies in their medical records. Nevertheless, there is quite a real possibility that the basic pathology (non-primary brain pathologies: hypotensive or hypovolemic shock, acute renal dysfunction, pulmonary oedema et cetera), combined with intensive care therapy administered (volume replacement, vasoactive medications) could have led to changes in the brain water content, thus changing the dielectric properties of the tissues in question. 

#### 4.4.3. Tissue Variability

Lastly, it is worth noting that there is a factor of variability of the measured dielectric properties even when the tissues are considered homogenous. Therefore, the measured permittivity results can vary greatly when measured at different locations on the same specimen. The measurement study by Schmid et al. [28] states that the observed extent of standard deviation is around 6% for the real part of the permittivity and between 8% and 10% for the imaginary part of the permittivity. It is thought to be caused by the combination of biological variances in the tissue and by the differences in tissue temperature at the measurement sites. Similarly, Gabriel [20] states that their results’ variability was in the range ±5%, and that the variation in tissue properties within a species may well exceed variations between species. That is an additional uncertainty factor that should be taken into consideration when comparing measurement results from seemingly identical specimens. The above applies not only to our measurement results, but also to all published results from the literature, which should always be taken with some reservation.

The average standard deviations for measured brain tissues are shown in Table 4.

Comparing our results with the other published ex vivo permittivity data, we find that our standard deviations generally match those previously reported. For example, Fornes-Leal et al. [49] reported the standard deviation of measured permittivity of healthy colon tissues, varying from 2.33 to 3.12 for $\varepsilon_{r}^{\prime \prime }$ and from 0.09 to 2.68 for *σ*. It is important to note the difference between *σ* and $\varepsilon_{r}^{\prime \prime }$ as *σ* has a smaller absolute variation in value than $\varepsilon_{r}^{\prime \prime }$. Another, more recent example is the ex vivo measurement of ovine hearts in [50], where the mean standard deviation for $\varepsilon_{r}^{\prime }$ varies from 1.98 to 9.4 and from 0.04 to 0.35 for *σ,* depending on the measured part of the heart. Again, one should note the difference between *σ* and $\varepsilon_{r}^{\prime \prime }$. Consequently, the reported standard deviation of our data is within the expected standard deviation caused by the natural variation of the biological tissues that is also present in other reported ex vivo measurements. 

## 5. Conclusions

The primary aim of this work was to report the measured dielectric permittivity of brain tissues, namely white matter, grey matter and cerebellum, in the microwave frequency range, from 0.5 to 18 GHz. Human, bovine and porcine brain samples were acquired and measured at the temperature of 25 °C. Human brain tissue permittivity is especially interesting for various medical uses; however, the measurement results in the literature are very scarce and dated. Therefore, the newly measured data certainly present a valuable addition to the knowledge about the human brain. The data containing permittivity values measured in this study are available as Supplementary Materials to this paper, to be freely used by other interested researchers.

Our results were compared with the published permittivity values for the corresponding tissues from the IT’IS database [21]. Furthermore, each species was compared with the suitable references for that species. The comparisons showed satisfactory overall agreement, while the observed differences led to important conclusions. The differences exist not only between our results and other references, but between the other references as well. This shows that the biological tissues exhibit variability in their permittivity within the same tissue type, which is a logical consequence of the natural variability of the tissue composition and physiological processes in the tissue specimen. This could be attributed to various factors, such as: age, health status, physiological condition, diet, postmortem sample handling etc. For most of the studies, these factors are mostly unknown or not disclosed. Therefore, each published data set, including our own, should be taken with some reservation. Accordingly, none of the references, not even those used here for comparison, can be considered as absolutely accurate data. 

Regarding the inter- and intraspecies variations of our own measurement results, the interspecies differences were noticeable, but comparable to the standard deviation within the individual species. 

In the course of this study, we used the opportunity to measure the same human white and grey matter samples over the course of three days, to examine the effects of tissue degradation on its permittivity. The trend suggested the loss of water content in the samples along the time course, despite keeping them in sealed containers in the refrigerator. This observation is in line with the expectations, and severely limits the usability of samples that are not freshly excised. 

An important conclusion can be made based on the comparison between the two human brains obtained from autopsies. The two brains showed noticeable differences in their permittivity, which could be attributed not only to their age difference and natural tissue variability, but also to the medical conditions of the deceased patients. After analyzing their medical records, we conclude that there is quite a real possibility that the basic non-brain-related pathology of the patients, combined with intensive care therapy, could have led to changes in brain water content, potentially more significant than any age-related water content changes, thus affecting the dielectric properties of the brain tissues. Investigation of this hypothesis is far beyond the scope of this study; however, this important consideration, not previously mentioned in the literature, should be taken into account when measuring the permittivity of human brain tissues, as most of them would be derived from hospital autopsies and not from healthy individuals. As an argument supporting this hypothesis, the three bovine brains obtained from healthy animals of similar age showed very similar permittivity values.

## Figures and Tables

**Table 1:** The number of measurement points per tissue and species type.

Species	Brain	Age	White Matter	Grey Matter	Cerebellum
**Human**	H1	50 years	14	15	15
	H2	84 years	18	10	5
**Bovine**	B1	Under 12 months	72	70	13
	B2		38	32	-
	B3		38	32	-
**Porcine**	P1	Unknown	38	40	-

**Table 2:** Average percent change in $\varepsilon_{r}^{\prime }$ and $\varepsilon_{r}^{\prime \prime }$ on day 2 and day 3 compared to day 1 for grey and white matter.

	Day 2		Day 3	
	$\varepsilon_{r}^{\prime }$	$\varepsilon_{r}^{\prime \prime }$	$\varepsilon_{r}^{\prime }$	$\varepsilon_{r}^{\prime \prime }$
**Grey Matter**	−11.19%	−15.29%	−28.55%	−32.81%
**White Matter**	8.97%	8.24%	−20.63%	−24.34%

**Table 3:** Single pole Cole–Cole parameters for measured brain tissues.

		$\varepsilon_{\infty }$	$\varepsilon_{s}$	$\alpha_{\;}$	$\sigma_{\;}$	$\tau_{\;}\left[ps\right]$
**Porcine Brain**	White Matter	5	31	0.09	0.47	5.4
	Grey Matter	3	45	0.05	0.8	6.2
**Bovine Brain**	White Matter	5	34	0.09	0.48	5.6
	Grey Matter	5	51	0.05	0.85	6.8
	Cerebellum	5	47	0.1	0.8	7.0
**Human Brain**	White Matter	2	36	0.13	0.5	5.8
	Grey Matter	5	51	0.05	0.9	7.1
	Cerebellum	3	47	0.08	0.83	6.5

**Table 4:** Average standard deviation for measured white matter, grey matter and cerebellum of all measured brains.

Brain Sample	White Matter		Grey Matter		Cerebellum	
	$\varepsilon_{r}^{\prime }$	$\varepsilon_{r}^{\prime \prime }$	$\varepsilon_{r}^{\prime }$	$\varepsilon_{r}^{\prime \prime }$	$\varepsilon_{r}^{\prime }$	$\varepsilon_{r}^{\prime \prime }$
**H1**	2.4	1.1	2.9	1.6	3.9	2.3
**H2**	8.1	4.5	3.1	1.9	2.1	1.5
**B1**	3.0	1.4	3.1	1.9	6.0	3.6
**B2**	2.1	0.9	1.7	0.9	-	-
**B3**	2.1	1.1	2.3	1.5	-	-
**P**	2.6	1.3	1.2	4.9	-	-

## Data Availability

The measured permittivity data sets are available as Supplementary Materials accompanying this paper.

## Supplementary Materials

The following supporting information can be downloaded at: https://www.mdpi.com/article/10.3390/diagnostics12112580/s1, the measured permittivity data sets, described in Section 3.7.
